# Water sorption behaviour of commercial furcellaran

**DOI:** 10.1016/j.heliyon.2022.e11056

**Published:** 2022-10-13

**Authors:** Kairit Eha, Aleksei Kaleda, Anne Menert, Katrin Laos

**Affiliations:** a Department of Chemistry and Biotechnology, Tallinn University of Technology, Akadeemia tee 15, 12618 Tallinn, Estonia; b Center of Food and Fermentation Technologies, Akadeemia tee 15A, 12618 Tallinn, Estonia; c Institute of Molecular and Cell Biology, University of Tartu, Ülikooli 18, 50090 Tartu, Estonia

**Keywords:** Furcellaran, Sorption isotherm, Modelling, Net isosteric heat of sorption

## Abstract

Water sorption isotherms are important tool for designing the technological processes and predicting stability and shelf life of food. The aim of the work was to determine the water sorption isotherms of commercial furcellaran at different temperatures (20, 35 and 50 °C) using a gravimetric method under different levels of relative humidity (19–95%). The experimental data obtained have been interpreted in the terms of various isotherm models and it was found that the best results were obtained with the Peleg model (P < 2.1%; RSME <0.01; R^2^ = 1.00). The obtained isotherms followed the type II pattern and, in all cases, the sorption capacity increased with increasing water activity and decreased with increasing temperature. The maximum net isosteric heat for adsorption and desorption was 938.9 and 988.6 J g ^−1^, respectively, at an equilibrium moisture content of 0.05 g_w_ g_db_^−1^. The net isosteric heat of sorption decreased exponentially with increasing moisture content and approached zero at 0.26 g_w_ g_db_^−1^ for adsorption and 0.32 g_w_ g_db_^−1^ for desorption. The developed mathematical relationships can be used to optimize the drying processes and storage conditions of furcellaran.

## Highlights

• Moisture sorption isotherms of furcellaran were determined.
• The optimal isotherm model for furcellaran is the Peleg model.
• An equation for the calculation of the net isosteric heat of the sorption of furcellaran was developed.

## 1 Introduction

Furcellaran is sulphated polysaccharide extracted from the red seaweed *Furcellaria lumbricalis*. It is a hybrid of kappa- and beta-carrageenan (Eha et al., 2021), and is used in the food industry as a gelling and stabilising agent.

The physical and chemical properties of biopolymers are strongly dependent on their temperature and moisture histories. The high temperatures during production of furcellaran and the interaction between water can change the hydrocolloid structure and may make it sensitive to ambient humidity. To understand the water sorption characteristics among biopolymers and atmosphere the water sorption isotherms are usually determined that describes the relationship between the equilibrium moisture content and water activity in certain temperature. These isotherms are important in order to optimise drying parameters, determine packaging requirements and estimate shelf life (Bazardeh and Esmaiili, 2014; Noriega et al., 2014; Bispo et al., 2015).

Several moisture sorption isotherm measurement techniques, such as vapour pressure manometric (Gasparik et al., 2013) and hygrometric (Demarchi et al., 2013; Pollatos et al., 2013), are available, although the most widely used and recommended is the static gravimetric method (Stepien et al., 2020; Arslan-Tontul, 2021).

Water sorption isotherms are generated during wetting (water adsorption) or drying (water desorption) of food. In the literature, different mathematical equations can be found to model isotherm data. Among the most common models used for describing sorption in food products are GAB (Guggenheim, Anderson & de Boer), Caurie, Henderson-Thompson, Oswin, Peleg, and Smith (Karatas and Arslan, 2022; Aguirre et al., 2021; Saleh et al., 2018; Panjagari et al., 2015).

The sorption process involves changes in the isosteric heat of desorption or adsorption. The isosteric heat of sorption estimates the moisture absorbed by solid particles (Mulet et al., 1999) and is defined as the difference between isosteric heat and the heat of vaporisation at the system temperature. It can be used for drying process design and provides information on the state of water molecules in food matrices (Tadapaneni et al., 2017).

Every food has its own special isotherms and net isosteric heat of sorption. There are studies available for the sorption properties of such hydrocolloids as carboxymethyl cellulose, guar, locust bean, tragacanth and xanthan gums (Torres et al., 2012). Pajpai and Pradeep (2013) investigated kappa-carrageenan sorption properties and found that the equilibrium moisture content data were best interpreted in terms of a GAB isotherm model. Later, Torres et al. (2018) found that kappa-, iota- and kappa/iota-hybrid carrageenan sorption data were well fitted using the Caurie model and the water sorption behaviour was influenced by molar fractions of repeating units and crystallinity. However, there is no available isotherm information on furcellaran. Understanding the water sorption characteristics of furcellaran is essential in industrial processing in order to optimise the drying process and ensure storage stability. So the aims of this work were to provide experimental data for the sorption characteristics of furcellaran, to find the best-fitting model to describe the sorption isotherms and to determine the net isosteric heat of sorption.

## 2 Materials and methods

### 2.1 Materials

The studied commercial furcellaran was an industrial product from AS EstAgar (Kärla, Estonia). Five saturated salt solutions (CH_3_COOK, Mg(NO_3_)_2_∗6H_2_O, NaCl, KCl and KNO_3_) were prepared to obtain constant relative humidity environments. All salt solutions were reagent grade.

### 2.2 Water sorption behaviour

#### 2.2.1 Sorption isotherms measurements

The water adsorption and desorption isotherms of furcellaran were determined in triplicate by static gravimetric method at the temperatures 20, 35 and 50 °C (±0.1 °C) for water activity range from 0.19 to 0.95 (Table 1). Selected temperature and water activity values are relevant with biopolymer storage. The samples for adsorption and desorption processes were placed into an environment of 8% relative humidity using NaOH and 100% relative humidity using H_2_O, respectively, until equilibrium was achieved (Torres et al., 2018). Then the dry and wet samples (0.5 ± 0.0001 g) for adsorption and desorption experiments were placed in Petri dishes and sealed hermetically into the jars containing different saturated salt solutions to provide atmospheres of desired relative humidity. The jars were placed in thermal cabinets at 20, 35, and 50 °C and the samples were weighed once a week until equilibrium was achieved. The constant weight (±0.0005 g) was established inaround three months.

**Table 1:** The water activities (*a*_w_) of saturated salt solutions at various temperatures (Greenspan, 1977).

	Salt solutions				
	CH_3_COOK	Mg(NO_3_)_2_∗6H_2_O	NaCl	KCl	KNO_3_
*a*_w_ at 20 °C	0.235	0.544	0.755	0.851	0.946
*a*_w_ at 35 °C	0.208	0.499	0.749	0.830	0.908
*a*_w_ at 50 °C	0.192	0.454	0.744	0.812	0.848

#### 2.2.2 Modelling of sorption isotherms

The experimental data regarding the sorption isotherms of furcellaran were fitted to the GAB, Caurie, Henderson-Thompson, modified Oswin, Peleg and Smith mathematical models. Table 2 shows equations for the used models in this study. The non-linear regression function from package “nls2” 0.2 for R 4.1.0 (R Foundation for Statistical Computing, Vienna, Austria) was used to fit the equations to experimental results and to estimate the parameters of the models.

**Table 2:** Sorption isotherm models used for furcellaran.

Model	Equation
GAB (Van der Berg and Bruin, 1981)	$X_{e}=\frac{a\;b\;c\;a_{w}}{\left(1-ca_{w}\right)\left(1-ca_{w}+bca_{w}\right)}$
Caurie (Castillo et al., 2003)	$X_{e}=\exp\left(a+ba_{w}\right)$
Henderson-Thompson (Thompson et al., 1968)	$X_{e}={\left[\frac{\ln\left(1-a_{w}\right)}{-a\left(T+c\right)}\;\right]}^{1/b}$
Modified Oswin (Chen, 2000)	$X_{e}=\left(a-bT\right){\left(\frac{a_{w}}{{1-a}_{w}}\right)}^{c}$
Peleg (Peleg, 1993)	$X_{e}=a{\left(a_{w}\right)}^{b\;}+c{\left(a_{w}\right)}^{d}$
Smith (Smith, 1947)	$X_{e}=a-b\;\left(\ln\left(1-a_{w}\right)\right)$

Where X_e_ is the equilibrium moisture content (g g^−1^ d.b.), *a*_w_ is the water activity, a, b, c, d are adjustable parameters and T is temperature (°C).

The goodness of fit of the different models to data was assessed by the mean relative percentage deviation modulus (P), the root mean square error (RMSE) and the coefficient of determination (R^2^), determined by using Eqs. (1), (2), and (3) (Bahloul et al., 2008).

(1) $$P=\frac{100}{N}\;\sum_{j=1}^{N}\;\left|\frac{{Xe}_{j\;cal}-{Xe}_{j\;\exp}}{{Xe}_{j\;\exp}}\right|$$

(2) $$RMSE=\sqrt{\sum_{j=1}^{N}\;{\frac{\left({Xe}_{j\;cal}-{Xe}_{j\;\exp}\right)}{N-n_{p}}}^{2}}$$

(3) $$R^{2}=\frac{S_{t}-SCE}{S_{t}}$$

where.

$S_{t}=\sqrt{\frac{\sum_{j=1}^{N}{\bar{(Xe}-{Xe}_{j})}^{2}}{n-1}}$$\bar{Xe}=\frac{\sum_{j=1}^{N}{Xe}_{j}}{N}$

$$SCE=\sum_{j=1}^{N}{\left({Xe}_{j\;cal}-{Xe}_{j\;\exp}\right)}^{2}$$

where Xe_j cal_ and Xe_j exp_ are calculated and experimental values of the equilibrium moisture content ($\bar{Xe}$), respectively. N is the number of data points and n_p_ is the number of free parameters in the model. SCE is the model sum of squares and S_t_ is the total sum of squares.

Values of P < 10%, RMSE ≤0.05 and R^2^ > 0.98 are indicative of a good fit (Sormoli and Langrish, 2015; Bastioglu et al., 2017).

#### 2.2.3 Net isosteric heat of sorption

The net isosteric heat of sorption (Q_st_) was calculated by applying equation derived from Clausius-Clapeyron equation to the moisture sorption data (Kammoun Bejar et al., 2012):

(4) $$\ln\left(a_{w}\right)=-\left(\frac{Q_{st}}{R}\right)\frac{1}{T}+K$$

where *a*_w_ is the water activity, Q_st_ is the net isosteric heat of sorption (J g^−1^), R is the water characteristic gas constant (0.4615 J g^−1^ K^−1^) and T the absolute temperature (K).

By plotting ln (*a*_w_) versus T^−1^ for the fixed moisture content of the material, linear relationships can be obtained, with slope which equals –Q_st_ ∗ R^−1^, from which the net isosteric heat of sorption can be calculated (Tsami, 1991).

The water activity values of the material for different temperatures were obtained using the equation that best fit the experimental moisture sorption data.

## 3 Results and discussion

### 3.1 Sorption isotherms

**Figure 1:**
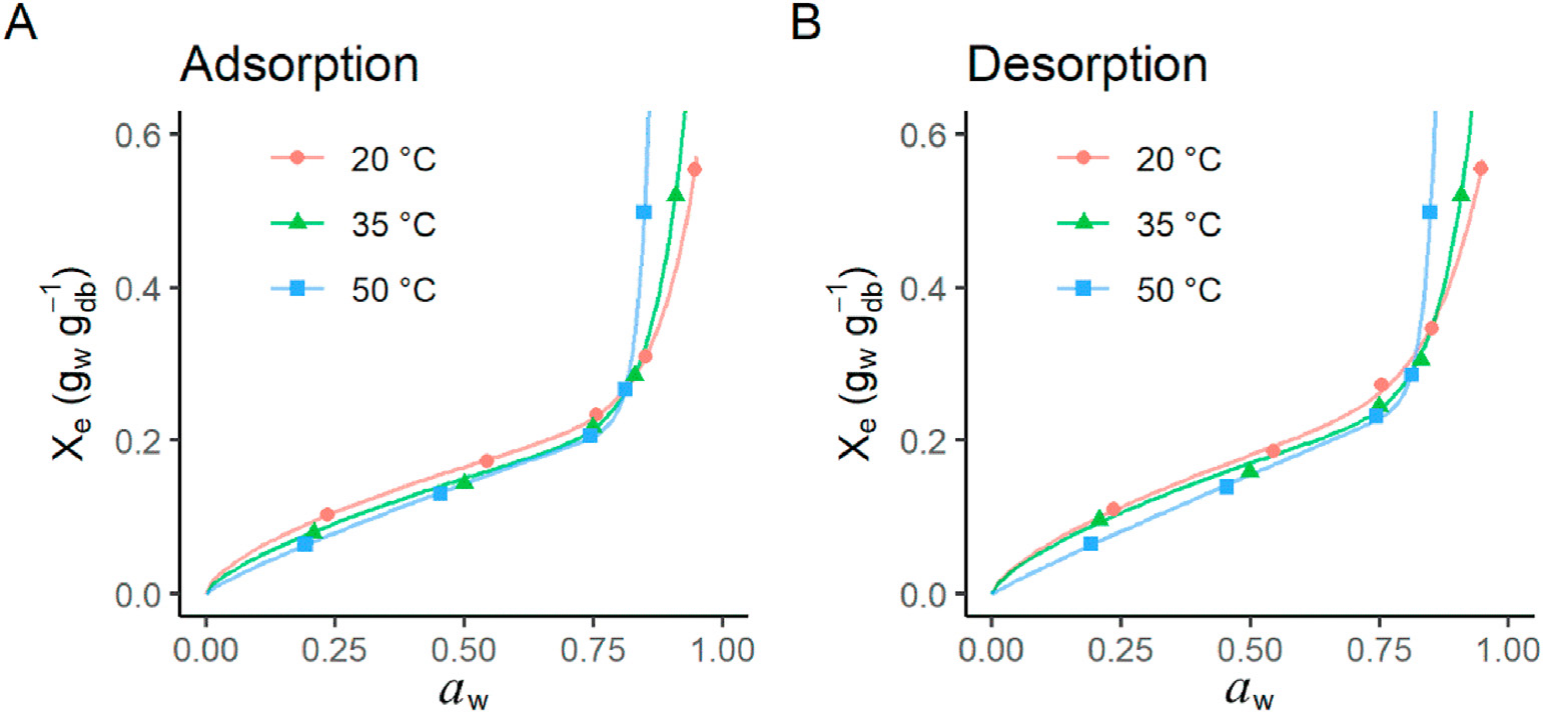
Water adsorption (A) and desorption (B) isotherms of furcellaran at different temperatures. X_e_ the equilibrium moisture content. Dots correspond to experimental data and lines to the Peleg model.

The moisture adsorption and desorption isotherms of furcellaran determined at 20, 35 and 50 °C are given in Figure 1. All sorption isotherms were Type II according to BET and IUPAC classifications and had sigmoidal shapes, which indicated multi-layers of sorption of water in a macroporous material (Sing et al., 1985). This behaviour is typical of agricultural and food products, such as melon seeds (Mallek-Ayadi et al., 2020), persimmon leaves (Martinez et al., 2014) and dairy products (Deshmukh et al., 2017). The equilibrium moisture content increased with increasing water activity at each temperature and decreased with increasing temperature at water activity <0.76, in all cases. This thermal behaviour may be explained by the fact that at higher temperatures the kinetic energy of water molecules increases, thereby increasing their distance and reducing the attractive forces between them. As a result, the sorption decreases with increasing water activity as the temperature increases (Bahloul et al., 2008).

A clear increase in moisture content can be seen in adsorption (Figure 1A) and correspondingly a decrease in moisture loss for desorption (Figure 1B) for water activity >0.76 at all temperatures. This phenomenon has been observed with foods rich in soluble solids such as sweetened yoghurt powder (Seth et al., 2018) and orange juice powder (Sormoli and Langrish, 2015). Soluble solids can bind water resulting the decrease of water activity. The intersecting of different isotherms can be observed. This is probably due to faster dissolution of soluble solids at higher temperatures. Also, more water binding sites in the furcellaran may be exposed due to the thermal effect.

**Figure 2:**
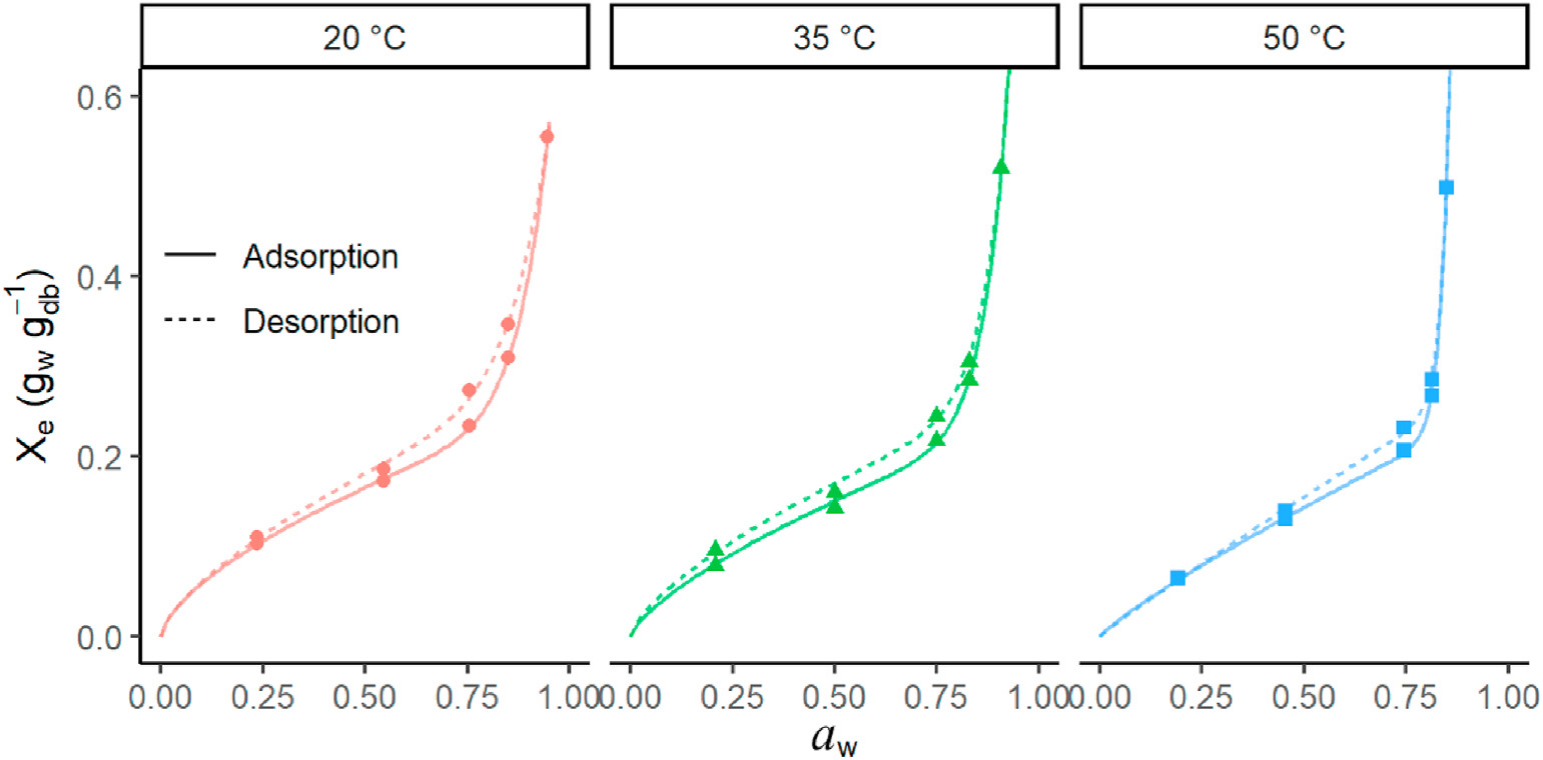
Sorption isotherms with a hysteresis between adsorption and desorption of furcellaran at different temperatures.

Small differences in hysteresis (the gap between adsorption and desorption) can be seen in comparing moisture adsorption and desorption isotherms at each temperature (Figure 2). However, the highest hysteresis was noticed at 20 °C and it was decreasing with increasing the temperature. This thermal effect may be caused by the change of the product structure and the solubility of some compounds in water (Bell and Labuza, 2000) and has been previously found for numerous food products, such as papayas (Udomkun et al., 2015), melon (*Cucumis melo*) peels (Mallek-Ayadi et al., 2022), and Algerian Bay leaves (*Laurus nobilis*) (Ouafi et al., 2015).

The parameter values and statistical results at 20, 35 and 50 °C for all models used for adsorption and desorption isotherms are shown in Table 3 and Table 4, respectively.

**Table 3:** Values of constants and statistical criteria of fitting the adsorption isotherms of furcellaran at various temperatures.

Adsorption isotherms								
Models names	T, °C	Parameters				P, %	RMSE	R^2^
		a	b	c	d			
GAB	20	0.074	109.611	0.914	–	7.6	0.03	0.99
	35	0.058	148.202	0.978	–	7.9	0.03	0.99
	50	0.066	73.220	0.996	–	19.2	0.08	0.87
Caurie	20	-3.791	3.273	–	–	23.5	0.06	0.90
	35	-4.488	4.127	–	–	29.7	0.06	0.89
	50	-4.896	4.733	–	–	38.4	0.09	0.80
Henderson Thompson	20	0.017	1.060	322.396	–	18.0	0.06	0.95
	35	0.011	0.788	366.182	–	26.5	0.07	0.92
	50	0.021	0.687	115.805	–	36.7	0.10	0.81
Modified Oswin	20	-1.821	-0.099	0.422	–	5.8	0.03	0.99
	35	-1.853	-0.057	0.565	–	12.5	0.05	0.96
	50	-2.362	-0.049	1.051	–	34.8	0.09	0.86
Peleg	20	0.257	0.637	0.626	12.917	0.6	0.00	1.00
	35	0.246	0.711	1.298	15.529	1.8	0.00	1.00
	50	0.260	0.856	149.854	38.293	1.1	0,00	1.00
Smith	20	0.031	0.168	–	–	13.1	0.04	0.96
	35	0.001	0.190	–	–	20.6	0.06	0.91
	50	0.001	0.207	–	–	24.3	0.09	0.78

P is mean relative percentage deviation modulus; RMSE is root mean square error; R^2^ is coefficient of determination; a, b, c, d are parameters of the equations; T is temperature (°C).

**Table 4:** Values of constants and statistical criteria of fitting the desorption isotherms of furcellaran at various temperatures.

Desorption isotherms								
Models names	T, °C	Parameters				P, %	RMSE	R^2^
		a	b	c	d			
GAB	20	0.096	64.942	0.870	–	3.5	0.02	0.99
	35	0.073	41.956	0.946	–	8.3	0.03	0.98
	50	0.069	108.357	0.997	–	17.6	0.07	0.91
Caurie	20	-3.290	2.765	–	–	15.4	0.05	0.94
	35	-3.653	3.180	–	–	22.4	0.06	0.90
	50	-4.037	3.721	–	–	28.2	0.08	0.83
Henderson Thompson	20	0.048	1.150	104.263	–	12.3	0.04	0.97
	35	0.017	0.964	242.226	–	21.3	0.06	0.92
	50	0.016	0.753	165.822	–	31.7	0.09	0.84
Modified Oswin	20	-2.035	-0.111	0.333	–	7.4	0.05	0.96
	35	-2.686	-0.081	0.547	–	11.8	0.04	0.97
	50	-2.646	-0.055	0.788	–	24.9	0.08	0.89
Peleg	20	0.289	0.677	0.492	10.416	1.9	0.01	1.00
	35	0.275	0.689	1.104	14.753	2.8	0.01	1.00
	50	0.298	0.936	148.924	38.990	1.7	0.01	1.00
Smith	20	0.054	0.165	–	–	5.4	0.02	0.99
	35	0.027	0.184	–	–	14.2	0.05	0.93
	50	0.001	0.213	–	–	21.0	0.08	0.83

P is mean relative percentage deviation modulus; RMSE is root mean square error; R^2^ is coefficient of determination; a, b, c, d are parameters of the equations; T is temperature (°C).

Two of the proposed models did not provide satisfactory fits to the experimental data. The P, RMSE and R^2^ values corresponding to the Caurie and Henderson-Thompson models showed that these models cannot be used to simulate the sorption data of furcellaran. Thus, the use of the Caurie model suggested by Torres et al. (2018) for hybrid carrageenans is not suitable for furcellaran. This may be due to their different material thermal histories. Friedenthal et al. (2020) showed that heat treatment of carrageenans will destabilise the galactans, opening more binding sites for water, and thus increasing water vapour absorption. Pajpai and Pradeep (2013) showed that the GAB model was the best for investigating the kappa-carrageenan sorption properties. Our results indicated that the GAB model was suitable for predicting the isotherm of furcellaran only at lower temperatures. However, the Peleg model was found to be the most appropriate model to describe both the adsorption and desorption curves (Figure 1) of furcellaran for different water activities possessing the lowest mean P (1.2% for adsorption and 2.1% for desorption), the lowest RSME (<0.01) and the highest coefficient of determination (R^2^ = 1.00). The Peleg model has been successfully applied for many foods, for example cowpea (*Vigna unguiculate* L. Walp) (Karatas and Arslan, 2022) and lactose-free probiotic powders (Dantas et al., 2021).

#### 3.1.1 Net isosteric heat of sorption

**Figure 3:**
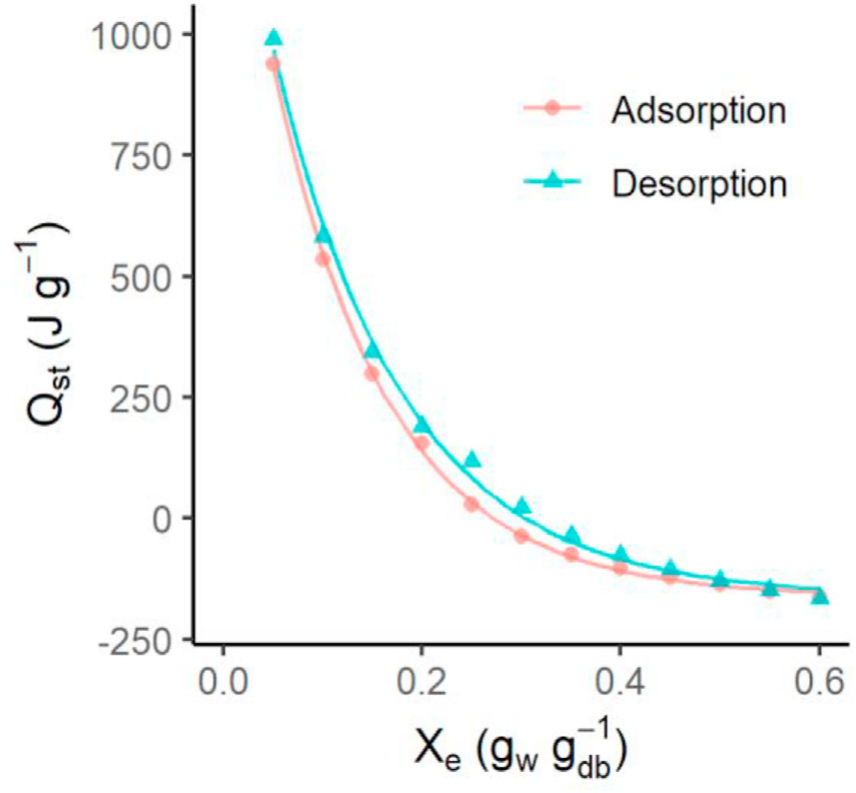
Net isosteric heat (Q_st_) of furcellaran for adsorption and desorption for different moisture contents (X_e_).

The experimental data of sorption isotherms were used to determine the net isosteric heat of the sorption of furcellaran. The Peleg model with Eq. (4) was used to obtain the values of the water activity at constant moisture content at each temperature (Figure 3).

The net isosteric heat decreases as the moisture content increases, showing that the energy needed to adsorb or remove the water in the furcellaran decreases with moisture content. It is known that the sorption occurs at first on the most active polar sites having the greatest interaction energy (Tsami, 1991). When increasing the water content, the sites will be occupied, sorption occurs at the less active sites and the net isosteric heat decreases. The net isosteric heat of desorption was slightly higher than that of adsorption, indicating that the energy required for the water desorption process was higher than that needed for the adsorption process. A similar trend has been observed in other studies for various products (Nayak et al., 2022; Tagnamas et al., 2021; Mbarek and Mihoubi, 2019). The maximum isosteric heats for adsorption and desorption were 938.9 and 988.6 J g^-1^, respectively at Xe 0.05 g_w_ g_db_^−1^.

The net isosteric heat of adsorption and desorption approached zero at 0.26 g_w_ g_db_^−1^ and 0.32 g_w_ g_db_^−1^, respectively. It shows that there is a critical moisture content where the net isosteric heat of adsorption and desorption is equal to the vaporisation of pure water, and the water molecules act as in the liquid state. So, at higher moisture content less energy is needed during material drying.

However, after a certain moisture content, the heat of sorption values became negative. This may be due to an increase in the moisture content by the dissolution of sugars and possibly of biopolymer at higher temperatures. Negative heat of sorption values has no physical meaning and is due to mathematical calculations (Kaymak-Ertekin and Gedik, 2004).

The net isosteric heat of the sorption of water in furcellaran can approximated mathematically by Eq. (5):

(5) $$Q_{st\;}=a*\exp\left(k*X_{e}\right)+b$$

For adsorption: P = 5.5; RMSE = 8.95; R^2^ = 0.999.

$$Q_{st\;}=1682.70\;\exp\;\left(-8.616*X_{e}\right)-161.40$$

For desorption: P = 15.65; RMSE = 20.74; R^2^ = 0.997.

$$Q_{st\;}=1655.41\;\exp\left(-7.632*X_{e}\right)-162.22$$

These mathematical equations can applied to predict the heat of the sorption of furcellaran for different moisture contents.

## 4 Conclusions

The moisture sorption behaviour of furcellaran exhibits the type II pattern. The sorption behaviour was found to decrease with temperature at fixed water activity with a crossover behaviour at water activity around 0.76. Hysteresis between the adsorption and desorption processes decreased with increasing temperature. The experimental sorption data of furcellaran was well expressed by the Peleg equation. The net isosteric heat of sorption was higher at low moisture contents, indicating that water molecules more strongly interact with furcellaran at the lower moisture content. The net isosteric heat of adsorption was lower than that of desorption and they negatively correlated with moisture content. The developed heat of the sorption equations will be useful in the simulation of furcellaran during drying process and storage. As drying also affects the molecular weight of the furcellaran, further work should be done to determine how this affects its water sorption properties.

## Declarations

### Author contribution statement

Kairit Eha: Performed the experiments; Analyzed and interpreted the data; Wrote the paper.

Aleksei Kaleda: Analyzed and interpreted the data; Wrote the paper.

Anne Menert, Katrin Laos: Conceived and designed the experiments; Wrote the paper.

### Funding statement

This work was supported by Eesti Teadusagentuur and European Regional Development Fund [RESTA12 & RESTA13].

### Data availability statement

Data will be made available on request.

### Declaration of interest's statement

The authors declare no conflict of interest.

### Additional information

No additional information is available for this paper.
